# Author Correction: Foundation model of neural activity predicts response to new stimulus types

**DOI:** 10.1038/s41586-026-10457-z

**Published:** 2026-04-08

**Authors:** Eric Y. Wang, Paul G. Fahey, Zhuokun Ding, Stelios Papadopoulos, Kayla Ponder, Marissa A. Weis, Andersen Chang, Taliah Muhammad, Saumil Patel, Zhiwei Ding, Dat Tran, Jiakun Fu, Casey M. Schneider-Mizell, MICrONS Consortium, Eric Y. Wang, Paul G. Fahey, Zhuokun Ding, Stelios Papadopoulos, Marissa A. Weis, Andersen Chang, Taliah Muhammad, Saumil Patel, Zhiwei Ding, Dat Tran, Jiakun Fu, Casey M. Schneider-Mizell, Nuno Maçarico da Costa, R. Clay Reid, Forrest Collman, Alexander S. Ecker, Jacob Reimer, Xaq Pitkow, Fabian H. Sinz, Andreas S. Tolias, R. Clay Reid, Forrest Collman, Nuno Maçarico da Costa, Katrin Franke, Alexander S. Ecker, Jacob Reimer, Xaq Pitkow, Fabian H. Sinz, Andreas S. Tolias

**Affiliations:** 1 Center for Neuroscience and Artificial Intelligence, Baylor College of Medicine, Houston, TX USA; 2 Department of Neuroscience, Baylor College of Medicine, Houston, TX USA; 3 Department of Ophthalmology, Byers Eye Institute, Stanford University School of Medicine, Stanford, CA USA; 4 Stanford Bio-X, Stanford University, Stanford, CA USA; 5 Wu Tsai Neurosciences Institute, Stanford University, Stanford, CA USA; 6 Institute of Computer Science and Campus Institute Data Science, University of Göttingen, Göttingen, Germany; 7 Allen Institute for Brain Science, Seattle, WA USA; 8 Max Planck Institute for Dynamics and Self-Organization, Göttingen, Germany; 9 Department of Electrical and Computer Engineering, Rice University, Houston, TX USA; 10 Institute for Bioinformatics and Medical Informatics, University of Tübingen, Tübingen, Germany; 11 Department of Electrical Engineering, Stanford University, Stanford, CA USA

**Keywords:** Extrastriate cortex, Network models, Computational models, Machine learning, Sensory processing

Correction to: *Nature* 10.1038/s41586-025-08829-y Published online 9 April 2025

To ensure accurate documentation of the implemented models, we clarify several architectural details in the Methods describing the Conv-LSTM and CvT-LSTM architectures. These clarifications are limited to the Methods description and do not affect the results or conclusions.

Perspective module: The Methods state that the pupil-position multilayer perceptron MLP uses an 8-dimensional hidden representation; however, in the implemented CvT-LSTM models, this module uses a 16-dimensional hidden representation.

Four-head ensemble: The Methods did not specify that the architecture used for the analyses is implemented as a four-head ensemble. In the implemented model, the modulation, core, and readout modules are independently parameterized across four heads (with shared perspective transform and readout grid), and predictions are obtained by averaging standardized log-responses cross heads.

Modulation module: The Methods state that the modulation network receives three behavioural inputs (treadmill velocity, pupil radius, and the derivative of pupil radius); however, in the implemented CvT-LSTM models, only treadmill velocity and pupil radius are used. In addition, the Methods describe the LSTM hidden and cell states as 8-dimensional; in the implemented models these states are 6-dimensional in the Conv-LSTM variant and 16-dimensional in the CvT-LSTM variant.

Core module (feedforward): The Methods state that the feedforward DenseNet blocks use the GELU nonlinearity; however, in the implemented Conv-LSTM models, the feedforward component uses ELU, whereas the CvT-LSTM models use GELU.

Core module (recurrent): In some Conv-LSTM model variants used in this work, the recurrent module additionally receives explicit spatial information about the visual stimulus. To do this, a spatial grid encoding the position of each feature-map element within the visual field is concatenated to the feedforward features and modulatory vector before entering the Conv-LSTM.

Core module (equations): In the editing process typographical errors were introduced in the equation blocks where before several terms an unnecessary curly brace, {, was added, and in several terms the convolutional operator ${(W}_{k}\ast )$ was incorrectly added as a superscript (${W}_{k}^{* }$).

For comparison, a Methods file with the edits highlighted is available as Supplementary Information accompanying this amendment. The text and equations have been amended in the HTML and PDF versions of this article.

## Supplementary information

Edits to Methods.

