# Supplementary material for: Foundation model of neural activity predicts response to new stimulus types
Source: Nature. 2025 Apr 9;640(8058):470–7. doi: 10.1038/s41586-025-08829-y (PMC11981942; doi:10.1038/s41586-025-08829-y)
Supplement: Supplementary file 1 — Reporting Summary [file 41586_2025_8829_MOESM1_ESM.pdf]

nature portfolio

Corresponding author(s): Andreas Tolias

Last updated by author(s): Jan 15, 2025

Reporting Summary

Nature Portfolio wishes to improve the reproducibility of the work that we publish. This form provides structure for consistency and transparency in reporting. For further information on Nature Portfolio policies, see our Editorial Policies and the Editorial Policy Checklist.

Statistics

For all statistical analyses, confirm that the following items are present in the figure legend, table legend, main text, or Methods section.

n/a	Confirmed	
☐	☒	The exact sample size (*n*) for each experimental group/condition, given as a discrete number and unit of measurement
☐	☒	A statement on whether measurements were taken from distinct samples or whether the same sample was measured repeatedly
☐	☒	The statistical test(s) used AND whether they are one- or two-sided<br>*Only common tests should be described solely by name; describe more complex techniques in the Methods section.*
☒	☐	A description of all covariates tested
☒	☐	A description of any assumptions or corrections, such as tests of normality and adjustment for multiple comparisons
☐	☒	A full description of the statistical parameters including central tendency (e.g. means) or other basic estimates (e.g. regression coefficient) AND variation (e.g. standard deviation) or associated estimates of uncertainty (e.g. confidence intervals)
☐	☒	For null hypothesis testing, the test statistic (e.g. *F*, *t*, *r*) with confidence intervals, effect sizes, degrees of freedom and *P* value noted<br>*Give P values as exact values whenever suitable.*
☒	☐	For Bayesian analysis, information on the choice of priors and Markov chain Monte Carlo settings
☒	☐	For hierarchical and complex designs, identification of the appropriate level for tests and full reporting of outcomes
☒	☐	Estimates of effect sizes (e.g. Cohen's *d*, Pearson's *r*), indicating how they were calculated

Our web collection on statistics for biologists contains articles on many of the points above.

Software and code

Policy information about availability of computer code

Data collection	For image acquisition, we used ScanImage 2017b. Stimuli were presented using PsychToolBox 3. The data collection process was automated with Labview.
Data analysis	We used DeepLabCut (2.0.5) for automatic tracking of the pupil. We used CaImAn (1.0) for automatic segmentation and deconvolution of calcium imaging data. Our custom built analysis pipeline (https://github.com/cajal/pipeline, https://github.com/cajal/foundation) also used general tools like Numpy (1.23.5), pandas (1.5.3), SciPy (1.10.1), statsmodels (0.13.5), scikit-learn (1.2.1), PyTorch (1.12.1), Matplotlib (3.7.0), seaborn (0.12.2), HoloViews (1.15.4), Ipyvolume (0.5.2), Jupyter (ipykernel: 6.21.2), MySQL (5.7.37), Docker (23.0.1), and Kubernetes (1.22.11). DataJoint (0.12.9) were used for storing and managing data.

For manuscripts utilizing custom algorithms or software that are central to the research but not yet described in published literature, software must be made available to editors and reviewers. We strongly encourage code deposition in a community repository (e.g. GitHub). See the Nature Portfolio guidelines for submitting code & software for further information.

Data

Policy information about availability of data

All manuscripts must include a data availability statement. This statement should provide the following information, where applicable:

- Accession codes, unique identifiers, or web links for publicly available datasets
- A description of any restrictions on data availability
- For clinical datasets or third party data, please ensure that the statement adheres to our policy

The MICrONS functional and structural data are available on BossDB (https://bossdb.org/project/microns-minnie, please also see https://www.microns-explorer.org/cortical-mm3 for details). The MICrONS foundation model is available on GitHub (https://github.com/cajal/fnn).

Research involving human participants, their data, or biological material

Policy information about studies with human participants or human data. See also policy information about sex, gender (identity/presentation), and sexual orientation and race, ethnicity and racism.

Reporting on sex and gender	*Use the terms sex (biological attribute) and gender (shaped by social and cultural circumstances) carefully in order to avoid confusing both terms. Indicate if findings apply to only one sex or gender; describe whether sex and gender were considered in study design; whether sex and/or gender was determined based on self-reporting or assigned and methods used. Provide in the source data disaggregated sex and gender data, where this information has been collected, and if consent has been obtained for sharing of individual-level data; provide overall numbers in this Reporting Summary. Please state if this information has not been collected. Report sex- and gender-based analyses where performed, justify reasons for lack of sex- and gender-based analysis.*
Reporting on race, ethnicity, or other socially relevant groupings	*Please specify the socially constructed or socially relevant categorization variable(s) used in your manuscript and explain why they were used. Please note that such variables should not be used as proxies for other socially constructed/relevant variables (for example, race or ethnicity should not be used as a proxy for socioeconomic status). Provide clear definitions of the relevant terms used, how they were provided (by the participants/respondents, the researchers, or third parties), and the method(s) used to classify people into the different categories (e.g. self-report, census or administrative data, social media data, etc.) Please provide details about how you controlled for confounding variables in your analyses.*
Population characteristics	*Describe the covariate-relevant population characteristics of the human research participants (e.g. age, genotypic information, past and current diagnosis and treatment categories). If you filled out the behavioural & social sciences study design questions and have nothing to add here, write "See above."*
Recruitment	*Describe how participants were recruited. Outline any potential self-selection bias or other biases that may be present and how these are likely to impact results.*
Ethics oversight	*Identify the organization(s) that approved the study protocol.*

Note that full information on the approval of the study protocol must also be provided in the manuscript.

Field-specific reporting

Please select the one below that is the best fit for your research. If you are not sure, read the appropriate sections before making your selection.

☒ Life sciences ☐ Behavioural & social sciences ☐ Ecological, evolutionary & environmental sciences

For a reference copy of the document with all sections, see nature.com/documents/nr-reporting-summary-flat.pdf

Life sciences study design

All studies must disclose on these points even when the disclosure is negative.

Sample size	No sample-size calculation was performed a priori. Sample sizes (number of connections tested) match or exceed previous studies of similar design.
Data exclusions	Of the 14 released MICrONS scans, one scan was excluded a priori from the study due to experimental issues (responses to some stimuli were not collected due to water running out from the objective). Duplicate detection was performed to identify neurons that were recorded more than once in experiments. Besides that, no neurons were exlcuded from model training or analysis.
Replication	The approach of using a foundation model core to fit new models of mice was replicated across 4 mice for evaluating predictive accuracy (Figure 3) and 3 mice for evaluating parametric tuning accuracy (Figure 4).
Randomization	No randomization of animal subjects was performed as our experimental design did not stratify into animal groups.
Blinding	No blinding is performed during data collection since our study did not include predefined experimental groups for sample allocation. The

Blinding	analysis is performed unblinded; however, the same computational methods were applied to all control and sample groups.

Reporting for specific materials, systems and methods

We require information from authors about some types of materials, experimental systems and methods used in many studies. Here, indicate whether each material, system or method listed is relevant to your study. If you are not sure if a list item applies to your research, read the appropriate section before selecting a response.

Materials & experimental systems

n/a	Involved in the study
☒	☐ Antibodies
☒	☐ Eukaryotic cell lines
☒	☐ Palaeontology and archaeology
☐	☒ Animals and other organisms
☒	☐ Clinical data
☒	☐ Dual use research of concern
☒	☐ Plants

Methods

n/a	Involved in the study
☒	☐ ChIP-seq
☒	☐ Flow cytometry
☒	☐ MRI-based neuroimaging

Animals and other research organisms

Policy information about studies involving animals; ARRIVE guidelines recommended for reporting animal research, and Sex and Gender in Research

Laboratory animals	For experiments excluding the MICrONS dataset in this manuscript: Three mice, Mus musculus, 78-86 days old at first experimental scan. Heterozygous for both Slc17a7-Cre (B6;129S-Slc17a7tm1.1(cre)Hze/J, Jackson Laboratory Strain # 023527) and Ai162 (B6.Cg-Igs7tm162.1(tetO-GCaMP6s,CAG-tTA2)Hze/J, Jackson Laboratory Strain # 031562). The MICrONS dataset was collected from a mouse of the same species and strain, 75 days old.
Wild animals	Study did not involve wild animals.
Reporting on sex	For new experiments in this manuscript: 6 Female, 8 Males. For MICrONS dataset, 1 Male. Animals were randomly recruited to the study with respect to sex. Analysis disaggregated for sex was not performed, due to low sample size and expected generalization of principles under study across genders.
Field-collected samples	Study did not involve samples collected from the field.
Ethics oversight	All procedures were approved by the Institutional Animal Care and Use Committee of Baylor College of Medicine.

Note that full information on the approval of the study protocol must also be provided in the manuscript.

Plants

Seed stocks	*Report on the source of all seed stocks or other plant material used. If applicable, state the seed stock centre and catalogue number. If plant specimens were collected from the field, describe the collection location, date and sampling procedures.*
Novel plant genotypes	*Describe the methods by which all novel plant genotypes were produced. This includes those generated by transgenic approaches, gene editing, chemical/radiation-based mutagenesis and hybridization. For transgenic lines, describe the transformation method, the number of independent lines analyzed and the generation upon which experiments were performed. For gene-edited lines, describe the editor used, the endogenous sequence targeted for editing, the targeting guide RNA sequence (if applicable) and how the editor was applied.*
Authentication	*Describe any authentication procedures for each seed stock used or novel genotype generated. Describe any experiments used to assess the effect of a mutation and, where applicable, how potential secondary effects (e.g. second site T-DNA insertions, mosiacism, off-target gene editing) were examined.*
