# Supplementary material for: Author Correction: Foundation model of neural activity predicts response to new stimulus types
Source: Nature. 2026 Apr 8;652(8111):E10. doi: 10.1038/s41586-026-10457-z (PMC13102710; doi:10.1038/s41586-026-10457-z)
Supplement: Supplementary file 1 — Edits to Methods. [file 41586_2026_10457_MOESM1_ESM.pdf]

nature portfolio

https://doi.org/10.1038/s41586-026-10457-z

Supplementary information

Author Correction: Foundation model of neural activity predicts response to new stimulus types

In the format provided by the authors and unedited

Neurophysiological experiments

MICrONS data in Fig. 5 was collected as described in the accompanying Article cite(MICrONS_Consortium2021-my), and data in Fig. 2a were collected as described cite(Sinz2018-sk). Data collection for all other figures is described below.

All procedures were approved by the Institutional Animal Care and Use Committee of Baylor College of Medicine. Fourteen mice (Mus musculus, 6 females, 8 males, age 2.2-4 months) expressing GCaMP6s in excitatory neurons via *Slc17a7*-Cre and Ai162 transgenic lines (recommended and shared by H. Zeng; JAX stock 023527 and 031562, respectively) were anaesthetized and a 4-mm craniotomy was made over the visual cortex of the right hemisphere as described previously cite(Reimer2014-ry, Froudarakis2014-lx). Mice were allowed at least five days to recover before experimental scans.

Mice were head-mounted above a cylindrical treadmill and 2P calcium imaging was performed using Chameleon Ti-Sapphire laser (Coherent) tuned to 920 nm and a large field of view mesoscope cite(Sofroniew2016-up) equipped with a custom objective (excitation NA 0.6, collection NA 1.0, 21 mm focal length). Laser power after the objective was increased exponentially as a function of depth from the surface according to: $P = P_0 \times e^{(z/L_z)}$, where P is the laser power used at target depth z, P_0 is the power used at the surface (not exceeding 20 mW), and L_z is the depth constant (220 μm). The highest laser output of 100 mW was used at approximately 420 μm from the surface.

The craniotomy window was leveled with regards to the objective with six degrees of freedom. Pixel-wise responses from a region of interest spanning the cortical window (>2,400 x 2,400 μm, 2-5 μm per pixel, between 100 and 220 μm from surface, > 2.47 Hz) to drifting bar stimuli were used to generate a sign map for delineating visual areas cite(Garrett2014-ki). Area boundaries on the sign map were manually annotated.

For 11 out of 15 scans (including four of the foundation cohort scans), our target imaging site was a 1,200 × 1,100 μm^2 area spanning L2-L5 at the conjunction of lateral V1 and 3 lateral higher visual areas: AL, LM, and RL. This resulted in an imaging volume that was roughly 50% V1 and 50% higher visual area. This target was chosen in order to mimic the area membership and functional property distribution in the MICrONS mouse cite(MICrONS_Consortium2021-my). Each scan was performed at 6.3 Hz, collecting eight 620 × 1,100 μm^2 fields per frame at 2.5 μm per pixel x-y resolution to tile a 1,200-1,220 × 1,100 μm^2 field of view at 4 depths (2 planes per depth, 20-40 μm overlap between coplanar fields). The four imaging planes were distributed across layers with at least 45 μm spacing, with 2 planes in L2/3 (depths: 170-200 μm and 215-250 μm), 1 in L4 (300-325 μm), and 1 in L5 (390-420 μm).

For the remaining four foundation cohort scans, our target imaging site was a single plane in L2/3 (depths 210-220 μm), spanning all visual cortex visible in the cortical window (typically including V1, LM, AL, RL, PM, and AM). Each scan was performed at 6.8-6.9 Hz, collecting four 630 μm width adjacent fields (spanning 2,430 μm region of interest, with 90 μm total overlap). Each field was a custom height (2,010-3,000 μm) in order to encapsulate visual cortex within that field. Imaging was performed at 3 μm per pixel.

Video of the eye and face of the mouse was captured throughout the experiment. A hot mirror (Thorlabs FM02) positioned between the left eye and the stimulus monitor was used to reflect an IR image onto a camera (Genie Nano C1920M, Teledyne Dalsa) without obscuring the visual stimulus. The position of the mirror and camera were manually calibrated per session and focused on the pupil. Field of view was manually cropped for each session. The field of view contained the left eye in its entirety, and was captured at ~20 Hz. Frame times were time stamped in the behavioural clock for alignment to the stimulus and scan frame times. Video was compressed using the Labview MJPEG codec with quality constant of 600 and stored the frames in AVI file.

Light diffusing from the laser during scanning through the pupil was used to capture pupil diameter and eye movements. A DeepLabCut model cite(Mathis2018-ti) was trained on 17 manually labelled samples from 11 mice to label each frame of the compressed eye video (intraframe only H.264 compression, CRF:17) with 8 eyelid points and 8 pupil points at cardinal and intercardinal positions. Pupil points with likelihood >0.9 (all 8 in 72-99% of frames per scan) were fit with the smallest enclosing circle, and the radius and centre of this circle was extracted. Frames with < 3 pupil points with likelihood >0.9 (<1.2% frames per scan), or producing a circle fit with outlier > 5.5x s.d. from the mean in any of the 3 parameters (centre x, centre y, radius, <0.2% frames per scan) were discarded (total <1.2% frames per scan). Gaps of ≤ 10 discarded frames were replaced by linear interpolation. Trials affected by remaining gaps were discarded (<18 trials per scan, <0.015%).

The mouse was head-restrained during imaging but could walk on a treadmill. Rostro-caudal treadmill movement was measured using a rotary optical encoder (Accu-Coder 15T-01SF-2000NV1ROC-F03-S1) with a resolution of 8,000 pulses per revolution, and was recorded at ~100 Hz in order to extract locomotion velocity. The treadmill recording was low-pass filtered with a Hamming window to remove high-frequency noise.

Monitor positioning and calibration

Visual stimuli were presented with Psychtoolbox in MATLAB to the left eye with a 31.0 x 55.2 cm (height x width) monitor (ASUS PB258Q) with a resolution of 1,080 x 1,920 pixels positioned 15 cm away from the eye. When the monitor is centred on and perpendicular to

the surface of the eye at the closest point, this corresponds to a visual angle of ~3.8° cm^{-1} at the nearest point and 0.7° cm^{-1} at the most remote corner of the monitor. As the craniotomy coverslip placement during surgery and the resulting mouse positioning relative to the objective is optimized for imaging quality and stability, uncontrolled variance in skull position relative to the washer used for head-mounting was compensated with tailored monitor positioning on a six-dimensional monitor arm. The pitch of the monitor was kept in the vertical position for all mice, while the roll was visually matched to the roll of the head beneath the headbar by the experimenter. In order to optimize the translational monitor position for centred visual cortex stimulation with respect to the imaging field of view, we used a dot stimulus with a bright background (maximum pixel intensity) and a single dark square dot (minimum pixel intensity). Randomly ordered dot locations drawn from either a 5 x 8 grid tiling the screen (20 repeats) or a 10 x 10 grid tiling a central square (approximately 90° width and height, 10 repeats), with each dot presentation lasting 200 ms. For five scans (four foundation cohort scans, one scan from Fig. 4), this dot-mapping scan targeted the V1-RL-AL-LM conjunction, and the final monitor position for each mouse was chosen in order to maximize inclusion of the population receptive field peak response in cortical locations spanning the scan field of view. In the remaining scans, the procedure was the same, but the scan field of view spanned all of V1 and some adjacent higher visual areas, and thus the final monitor position for each mouse was chosen in order to maximize inclusion of the population receptive field peak response in cortical locations corresponding to the extremes of the retinotopic map. In both cases, the yaw of the monitor visually matched to be perpendicular to and 15 cm from the nearest surface of the eye at that position.

A photodiode (TAOS TSL253) was sealed to the top left corner of the monitor, and the voltage was recorded at 10 kHz and timestamped with a 10 MHz behavior clock. Simultaneous measurement with a luminance meter (LS-100 Konica Minolta) perpendicular to and targeting the centre of the monitor was used to generate a lookup table for linear interpolation between photodiode voltage and monitor luminance in cd m^{-2} for 16 equidistant values from 0-255, and 1baseline value with the monitor unpowered.

At the beginning of each experimental session, we collected photodiode voltage for 52 full-screen pixel values from 0 to 255 for 1-s trials. The mean photodiode voltage for each trial was collected with an 800-ms boxcar window with 200-ms offset. The voltage was converted to luminance using previously measured relationship between photodiode voltage and luminance and the resulting luminance versus voltage curve was fit with the function $L = B + A \cdot P^{\gamma}$ where L is the measured luminance for pixel value P, and the median γ of the monitor was fit as 1.73 (range 1.58 - 1.74). All stimuli were shown without linearizing the monitor (that is, with monitor in normal gamma mode).

During the stimulus presentation, display frame sequence information was encoded in a three-level signal, derived from the photodiode, according to the binary encoding of the display frame (flip) number assigned in order. This signal underwent a sine convolution, allowing for local peak detection to recover the binary signal together with its behavioural time stamps. The encoded binary signal was reconstructed for >96% of the flips. Each flip was time stamped by a stimulus clock (MasterClock PCIe-OSC-HSO-2 card). A linear fit was applied to the flip time stamps in the behavioural and stimulus clocks, and the parameters of that fit were used to align stimulus display frames with scanner and camera frames. The mean photodiode voltage of the sequence encoding signal at pixel values 0 and 255 was used to estimate the luminance range of the monitor during the stimulus, with minimum values of approximately 0.005 - 1 cd m^{-2} and maximum values of approximately 8.0 - 11.5 cd m^{-2}.

Scan and behavioral data preprocessing

Scan images were processed with the CAIMAN pipeline cite(Giovannucci2019-gw), as described cite(MICrONS_Consortium2021-my), to produce the spiking activity neurons at the scan rate of 6.3-6.9 Hz. The neuronal and behavioural (pupil and treadmill) activity were resampled via linear interpolation to 29.967 Hz, to match the presentation times of the stimulus video frames.

Stimulus composition

We used dynamic libraries of natural movies cite(KarpathyCVPR14) and directional pink noise (Monet) as described cite(MICrONS_Consortium2021-my), and the static natural image library as described in Walker et al. cite(Walker2019-oq).

Dynamic Gabor filters were generated as described, cite(st-gabor).

We used a spatial envelope that had a s.d. of approximately 16.4° in the centre of the monitor. A 10-s trial consisted of 10 Gabor filters (each lasting 1 s) with randomly sampled spatial positions, directions of motion, phases, spatial and temporal frequencies.

Random dot kinematograms were generated as described, cite(morrone_cortical_2000). The radius of the dots was approximately 2.6° in the centre of the monitor. Each 10-s trial contained 5 patterns of optical flow, each lasting 2 s. The patterns were randomly sampled in terms of type of optical flow (translation: up/down/right/left, radial: in/out, rotation: clockwise/anticlockwise), and coherence of random dots (50%, 100%).

The stimulus compositions of the MICrONS recording sessions is described the accompanying Article, cite(MICrONS_Consortium2021-my). For all other recording session, the stimulus compositions are listed in Extended Data Table 1.

Neural network architecture

Our model of the visual cortex is an ANN composed of four modules: perspective, behaviour, core, and readout. These modules are described in the following sections.

Perspective module

The perspective module uses ray tracing to infer the perspective or retinal activation of a mouse at discrete time points from two input variables: stimulus (video frame) and eye position (estimated centre of pupil, extracted from the eye tracking camera). To perform ray tracing, we modelled the following physical entities: (1) topography and light ray trajectories of the retina; (2) rotation of the retina; (3) position of the monitor relative to the retina; (4) intersection of the light rays of the retina and the monitor.

(1) We modelled the retina as a uniform 2D grid mapped onto a 3D sphere via an azimuthal equidistant projection (Extended Data Fig. 1a). Let θ and ϕ denote the polar coordinates (radial and angular, respectively) of the 2D grid. The following mapping produces a 3D light ray for point (θ, ϕ) of the modelled retina:

$$\boldsymbol{l}(\theta, \phi): \begin{bmatrix} \theta \\ \varphi \end{bmatrix} \mapsto \begin{bmatrix} sin\theta \; cos\varphi \\ sin\theta \; sin\varphi \\ cos\theta \end{bmatrix}.$$

(2) We used pupil tracking data to infer the rotation of the occular globe and the retina.

At each time point t, a multilayer perceptron (MLP with 3 layers and 8 hidden units per layer for the Conv-LSTM architecture and 3 layers and 16 hidden units per layer for the CvT-LSTM architecture) is used to map the pupil position onto the 3 ocular angles of rotation:

$$MLP: \begin{bmatrix} p_{xt} \\ p_{yt} \end{bmatrix} \mapsto \begin{bmatrix} \widehat{\theta_{xt}} \\ \widehat{\theta_{yt}} \\ \widehat{\theta_{zt}} \end{bmatrix},$$

where the p_{xt}, p_{yt} are the x, y coordinates of the pupil center in the frame of the tracking camera at time t, and $\widehat{\theta_{xt}}, \widehat{\theta_{yt}}, \widehat{\theta_{zt}}$ are the estimated angles of rotation of about the x (adduction-abduction), y (elevation-depression), z (intorsion-extorsion) axes of the occular globe at time t.

Let $\boldsymbol{R}_x, \boldsymbol{R}_y, \boldsymbol{R}_z \in \mathbb{R}^{3\times3}$ denote rotation matrices about x, y and z axes. Each light ray of the retina $l(\theta, \phi)$ is rotated by the occular angles of rotation:

$$\hat{\boldsymbol{l}}(\theta, \phi, t) = \boldsymbol{R}_z(\widehat{\theta_{zt}})\boldsymbol{R}_y(\widehat{\theta_{yt}})\boldsymbol{R}_x(\widehat{\theta_{xt}})\boldsymbol{l}(\theta, \phi)$$

producing $\hat{l}(\theta, \phi, t) \in \mathbb{R}^3$, the ray of light for point (θ, ϕ) of the retina at time t, which accounts for the gaze of the mouse and the rotation of the occular globe.

(3) We modelled the monitor as a plane with six degrees of freedom: three for translation and three for rotation. Translation of the monitor plane relative to the retina is parameterized by $m_0 \in \mathbb{R}^3$. Rotation is parameterized by angles $\overline{\theta_x}, \overline{\theta_y}, \overline{\theta_z}$:

$$[\boldsymbol{m}_x\ \boldsymbol{m}_y\ \boldsymbol{m}_z] = \boldsymbol{R}_z(\overline{\theta_z})\boldsymbol{R}_y(\overline{\theta_y})\boldsymbol{R}_x(\overline{\theta_x}),$$

where $m_x, m_y, m_z \in \mathbb{R}^3$ are the horizontal, vertical, and normal unit vectors of the monitor.

(4) We computed the line-plane intersection between the monitor plane and $\hat{l}(\theta, \phi, t)$, the gaze-corrected trajectory of light for point ij of the retina at time t:

$$\boldsymbol{m}(\theta, \phi, t) = \frac{\boldsymbol{m}_0 \cdot \boldsymbol{m}_z}{\hat{\boldsymbol{l}}(\theta, \phi, t) \cdot \boldsymbol{m}_z}\hat{\boldsymbol{l}}(\theta, \phi, t),$$

where $m(\theta, \phi, t)$ is the point of intersection between the monitor plane and the light ray $\hat{l}(\theta, \phi, t)$. This is projected onto the monitor's horizontal and vertical unit vectors:

$$m^x(\theta, \phi, t) = (\boldsymbol{m}(\theta, \phi, t) - \boldsymbol{m}_0) \cdot \boldsymbol{m}_x,$$

$$m^y(\theta, \phi, t) = (\boldsymbol{m}(\theta, \phi, t) - \boldsymbol{m}_0) \cdot \boldsymbol{m}_y$$

yielding $m^x(\theta, \phi, t)$ and $m^y(\theta, \phi, t)$, the horizontal and vertical displacements from the centre of the monitor/stimulus (Extended Data Fig. 1b). To produce inferred activation of the retinal grid at (θ, ϕ, t), we performed bilinear interpolation of the stimulus at the four pixels surrounding the line-plane intersection at $m^x(\theta, \phi, t), m^y(\theta, \phi, t)$.

<u>Modulation module</u>

The modulation module is a small long short-term memory (LSTM) network cite(lstm) that transforms behavioural variables--- that is, locomotion and pupil size--- and previous states of the network, to produce dynamic representations of the behavioural state and arousal of the mouse.

$$\text{LSTM:} \begin{bmatrix} r_t \\ p_t \\ p'_t \end{bmatrix}, \boldsymbol{h}^m_{t-1}, \boldsymbol{c}^m_{t-1} \mapsto \boldsymbol{h}^m_t, \boldsymbol{c}^m_t,$$

where r is the running/treadmill speed, p is the pupil diameter, p' is the instantaneous change in pupil diameter, and $h^m, c^m \in \mathbb{R}^6$ are the "hidden" and "cell" state vectors of the modulation LSTM network.

In the CvT-LSTM architectures, the modulation LSTM has the form:

$$\text{LSTM}: \begin{bmatrix} r_t \\ p_t \end{bmatrix}, \boldsymbol{h}_{t-1}^m, \boldsymbol{c}_{t-1}^m \mapsto \boldsymbol{h}_t^m, \boldsymbol{c}_t^m,$$

where $h^m, c^m \in \mathbb{R}^{16}$

The hidden state vector h^m is tiled across space to produce modulation feature maps H_t^m:

$\boldsymbol{h}_t^m \in \mathbb{R}^C \to H_t^m \in \mathbb{R}^{C\times H\times W}$,

where C, H, W denote channel, height, and width, respectively, of the feature maps. These feature maps H_t^m serve as the modulatory inputs into the recurrent portion of the core module at time t.

Core module

The core module---comprised of feedforward and recurrent components---transforms the inputs from the perspective and modulation modules to produce feature representations of vision modulated by behavior.

First, the feedforward module transforms the visual input provided by the perspective module. For this we used DenseNet architecture cite(huang_densely_2018) with three blocks. Each block contains two layers of 3D (spatiotemporal) convolutions followed by a nonlinearity (ELU for Conv-LSTM and GeLU for CvT-LSTM architectures cite(hendrycks_gaussian_2020)) and dense connections between layers. After each block, spatial pooling was performed to reduce the height and width dimensions of the feature maps. To enforce causality, we shifted the 3D convolutions along the temporal dimension, such that no inputs from future time points contributed to the output of the feedforward module.

Next, the recurrent module transforms the visual and behavioral information provided by the feedforward and modulation modules, respectively, through a group of recurrent cells. We used a convolutional LSTM (Conv-LSTM) cite(conv-lstm) as the architecture for each recurrent cell. For each cell c, the formulation of the Conv-LSTM is shown below:

$$\boldsymbol{X}_t^c = W_1 * \boldsymbol{H}_t^f + W_1 * \boldsymbol{H}_t^m + \sum_{c'} W_1 * \boldsymbol{H}_{t-1}^{c'},$$

$$\boldsymbol{I}_t^c = \sigma(W_3 * \boldsymbol{X}_t^c + W_3 * \boldsymbol{H}_{t-1}^c + \boldsymbol{b}_i^c),$$

$$\boldsymbol{O}_t^c = \sigma(W_3 * \boldsymbol{X}_t^c + W_3 * \boldsymbol{H}_{t-1}^c + \boldsymbol{b}_o^c),$$

$$\boldsymbol{F}_t^c = \sigma\left(W_3 * \boldsymbol{X}_t^c + W_3 * \boldsymbol{H}_{t-1}^c + \boldsymbol{b}_f^c\right),$$

$$\boldsymbol{G}_t^c = \tanh\left(W_3 * \boldsymbol{X}_t^c + W_3 * \boldsymbol{H}_{t-1}^c + \boldsymbol{b}_g^c\right),$$

$$\boldsymbol{C}_t^c = \boldsymbol{F}_t^c \odot \boldsymbol{C}_{t-1}^c + \boldsymbol{I}_t^c \odot \boldsymbol{G}_t^c,$$

$$\boldsymbol{H}_t^c = \boldsymbol{O}_t^c \odot \tanh(\boldsymbol{C}_t^c),$$

where σ denotes the sigmoid function, ⊙ denotes the Hadarmard product, and $\mathrm{W}_k *$ denotes a 2D spatial convolution with a $k \times k$ kernel. $\boldsymbol{H}_t^f, \boldsymbol{H}_t^m$ are the feedforward and modulation outputs, respectively, at time t, and $H_{t-1}^{c'}$ is the hidden state of an external cell c' at time $t-1$. For cell c at time t, X_t^c, C_t^c, H_t^c are the input, cell, and hidden states, respectively, and I_t^c, O_t^c , $\boldsymbol{F}_t^c, \boldsymbol{G}_t^c$ are the input, output, forget, and cell gates.

To produce the output of the core network, the hidden feature maps of the recurrent cells are concatenated along the channel dimension:

$$H_t = \text{Concatenate}(H_t^{c=1}, H_t^{c=2}, \dots).$$

In some Conv-LSTM model variants used in this work, the recurrent module additionally receives explicit spatial information about the visual stimulus. To do this, a spatial grid encoding the position of each feature map element within the visual field is concatenated to the feedforward features and modulatory vector before entering the Conv-LSTM.

Given the recent popularity and success of transformer networks cite(vaswani_attention_2023), we explored whether adding the attention mechanism to our network would improve performance. We modified the Conv-LSTM architecture to incorporate the attention mechanism from the convolutional vision transformer (CvT) cite(wu_cvt_2021). This recurrent transformer architecture, which we name CvT-LSTM, is described as follows:

$$\boldsymbol{X}_t^c = W_1 * \boldsymbol{H}_t^f + W_1 * \boldsymbol{H}_t^m + \sum_{c'} W_1 * \boldsymbol{H}_{t-1}^{c'},$$

$$\boldsymbol{Z}_t^c = W_3 * \boldsymbol{X}_t^c + W_3 * \boldsymbol{H}_{t-1}^c,$$

$$\boldsymbol{Q}_t^c = W_1 * \boldsymbol{Z}_t^c,$$

$$\boldsymbol{K}_t^c = W_1 * \boldsymbol{Z}_t^c,$$

$$\boldsymbol{V}_t^c = W_1 * \boldsymbol{Z}_t^c,$$

$$\boldsymbol{A}_t^c = \text{Attention}(\boldsymbol{Q}_t^c, \boldsymbol{K}_t^c, \boldsymbol{V}_t^c),$$

$$\boldsymbol{I}_t^c = \sigma(W_1 * \boldsymbol{A}_t^c + W_1 * \boldsymbol{Z}_t^c + \boldsymbol{b}_i^c),$$

$$\boldsymbol{O}_t^c = \sigma(W_1 * \boldsymbol{A}_t^c + W_1 * \boldsymbol{Z}_t^c + \boldsymbol{b}_o^c),$$

$$F_t^c = \sigma(W_1 * A_t^c + W_1 * Z_t^c + b_f^c),$$

$$G_t^c = \tanh(W_1 * A_t^c + W_1 * Z_t^c + b_g^c),$$

$$C_t^c = F_t^c \odot C_{t-1}^c + I_t^c \odot G_t^c,$$

$$H_t^c = O_t^c \odot \tanh(C_t^c)$$

where attention is performed over query Q_t^c, key K_t^c, and value V_t^c spatial tokens, which are produced by convolutions of the feature map Z_t^c. The technique of using convolutions with the attention mechanism was introduced with CvT cite(wu_cvt_2021), and here we extend it by incorporating it into a recurrent LSTM architecture (CvT-LSTM).

We compare the performance of Conv-LSTM vs CvT-LSTM recurrent architecture in Extended Data Fig. 5. When trained on the full amount of data, Conv-LSTM performs very similarly to CvT-LSTM. However, Conv-LSTM outperforms CvT-LSTM when trained on restricted data (for example, 4 min of natural videos). This was consistent for all stimulus domains that were used to test model accuracy -- natural videos (Extended Data Fig. 5a), natural images (Extended Data Fig. 5b), drifting Gabor filters (Extended Data Fig. 5c), flashing Gaussian dots (Extended Data Fig. 5d), directional pink noise (Extended Data Fig. 5e), and random dot kinematograms (Extended Data Fig. 5f). The performance difference under data constraints may be due a better inductive bias of the Conv-LSTM. Alternatively, it could be due to a lack of optimization of the CvT-LSTM hyperparameters, and a more extensive hyperparameter search may yield better performance.

Readout module

The readout module maps the core's outputs onto the activity of individual neurons. For each neuron, the readout parameters are factorized into two components: spatial position and feature weights. For a neuron n, let $p^n \in \mathbb{R}^2$ denote the spatial position (x, y), and let $w^n \in \mathbb{R}^C$ denote the feature weights for that neuron, with $C = 512$ being the number of channels in the core module's output. To produce the response of that neuron n at time t, the following readout operation is performed:

$$\boldsymbol{h}_t^n = \text{Interpolate}(\boldsymbol{H}_t, \boldsymbol{p}^n),$$

$$r_t^n = \exp(\boldsymbol{h}_t^n \cdot \boldsymbol{w}^n + b^n),$$

where $h_t^n \in \mathbb{R}^C$ is a feature vector that is produced via bilinear interpolation of the core network's output $H_t \in \mathbb{R}^{C\times H\times W}$ (channels, height, width), interpolated at the spatial position p^n. The feature vector h_t^n is then combined with the feature weights w^n and a scalar bias b^n to produce the response r_t^n of neuron n at time t.

Due to the bilinear interpolation at a single position, each neuron only reads out from the core's output feature maps within a 2×2 spatial window. While this adheres to the functional property of spatial selectivity exhibited by neurons in the visual cortex, the narrow window limits exploration of the full spatial extent of features during model training. To facilitate the spatial exploration of the core's feature maps during training, for each neuron n, we sampled the readout position from a 2D Gaussian distribution: $p^n \sim \mathcal{N}(\mu^n, \Sigma^n)$. The parameters of the distribution μ^n, Σ^n (mean, covariance) were learned via the reparameterization trick, cite(kingma-vae-2013).

We observed empirically that the covariance Σ^n naturally decreased to small values by the end of training, meaning that the readout converged on a specific spatial position. After training, and for all testing purposes, we used the mean of the learned distribution μ^n as the single readout position $\boldsymbol{p}^n$ for neuron n.

In Extended Data Fig. 6, we examine the stability of the learned readout feature weights across different recording sessions. Due to the overlap between imaging planes, some neurons were recorded multiple times within the MICrONS volume. We found that the readout feature weights of the same neuron were more similar than feature weights of different neurons that were close in proximity, indicating that the readout feature weights of our model offer an identifying barcode of neuronal function that is stable across experiments.

Four-head ensemble

In the implemented models, the modulation, core, and readout modules are independently parameterized across four heads (with shared perspective transform and readout grid), and predictions are obtained by averaging standardized log-responses across heads.

Model training

The perspective, behaviour, core, and readout modules were assembled together to form a model that was trained to match the recorded dynamic neuronal responses from the training dataset. Let y_t^i be the recorded in vivo response, and let r_t^i be the predicted in silico response of neuron i at time t. The ANN was trained to minimize the Poisson negative-log likelihood loss, $\sum_{it} r_t^i - y_t^i \log(r_t^i)$, via stochastic gradient descent with Nesterov momentum cite(pmlr-v28-sutskever13-nesterov). The ANN was trained for 200 epochs with a learning rate schedule that consisted of a linear warm-up in the first 10 epochs, cosine decay cite(cosine-lr) for 90 epochs, followed by a warm restart and cosine decay for the remaining 100 epochs. Each epoch consisted of 512 training iterations / gradient descent steps. We used a batch size of 5, and each sample of the batch consisted of 70 frames (2.33 s) of stimulus, neuronal, and behavioural data.

Model hyperparameters

We used a grid search to identify architecture and training hyperparameters. Model performances for different hyperparameters were evaluated using a preliminary set of mice. After optimal hyperparameters were identified, we used the same hyperparameters to train models on a separate set of mice, from which the figures and results were produced. There was no overlap in the mice and experiments used for hyperparameter search and the mice and experiments used for the final models, results, and figures. This was done to prevent overfitting and to ensure that model performance did not depend on hyperparameters that were fit specifically for certain mice.

Model testing

We generated model predictions of responses to stimuli that were included in the experimental recordings but excluded from model training. To evaluate the accuracy of model predictions, for each neuron we computed the correlation between the mean in silico and in vivo responses, averaged over stimulus repeats. The average in vivo response aims to estimate the true expected response of the neuron. However, when the in vivo response is highly variable and there are a limited number of repeats, this estimate becomes noisy. To account for this, we normalized the correlation by an upper bound proposed by Schoppe et al. cite(Schoppe2016). Using $\overline{\cdot}$ to denote average over trials or stimulus repeats, the normalized correlation CC_{norm} is defined as follows:

$$CC_{norm} = \frac{CC_{abs}}{CC_{max}},$$

$$CC_{abs} = \frac{\mathrm{Cov}(\bar{r}, \bar{y})}{\sqrt{\mathrm{Var}(\bar{r})\mathrm{Var}(\bar{y})}},$$

$$CC_{max} = \sqrt{\frac{N\mathrm{Var}(\bar{y}) - \overline{\mathrm{Var}(y)}}{(N-1)\mathrm{Var}(\bar{y})}},$$

where r is the in silico response, y is the in vivo response, and N is the number of trials. CC_{abs} is the Pearson correlation coefficient between the average in silico and in vivo responses. CC_{max} is the upper bound of achievable performance given the ~~the~~ in vivo variability of the neuron and the number of trials.

Parametric tuning

To estimate parametric tuning, we presented parametric stimuli to the mice and the models. Specifically, we used directional pink noise parameterized by direction/orientation and flashing Gaussian blobs parameterized by spatial location. Orientation, direction, and

spatial tuning were computed from the recorded responses from the mice and the predicted responses from the models. This resulted in analogous in vivo and in silico estimates of parametric tuning for each neuron. The methods for measuring the tuning to orientation, direction, and spatial location are explained in the following sections.

Orientation and Direction tuning

We presented 16 angles of directional pink noise, uniformly distributed between $[0,2\pi)$. Let $\overline{r}_\theta$ be the mean response of a neuron to the angle θ, averaged over repeated presentations of the angle. The OSI and DSI were computed as

$$OSI = \frac{\left|\sum_\theta \overline{r_\theta} e^{i2\theta}\right|}{\sum_\theta \overline{r_\theta}},$$

$$DSI = \frac{\left|\sum_\theta \overline{r_\theta} e^{i\theta}\right|}{\sum_\theta \overline{r_\theta}},$$

That is, the normalized magnitude of the first and second Fourier components.

To determine the parameters for orientation and direction tuning, we used the following parametric model:

$$f(\theta|\mu,\kappa,\alpha,\beta,\gamma) = \alpha e^{\kappa\cos(\theta-\mu)} + \beta e^{\kappa\cos(\theta-\mu+\pi)} + \gamma,$$

which is a mixture of two von Mises functions with amplitudes α and β, preferred directions μ and $\mu+\pi$, and dispersion κ, plus a baseline offset of γ. The preferred orientation is the angle that is orthogonal to μ between $[0,\pi]$, that is, $(\mu+\pi/2)$ mod π. To estimate the parameters μ, $\kappa, \alpha,$ β, γ that best fit the neuronal response, we performed least squares optimization, minimizing $\sum_\theta (f(\theta \mid \mu, \kappa, \alpha, \beta, \gamma) - r_\theta)^2$.

Parameters were estimated via least square optimization for both the in vivo and in silico responses. Let $\hat{\mu}$ and $\overline{\mu}$ be the angles of preferred directions estimated from in vivo, in silico responses, respectively. The angular distances between the in vivo and in silico estimates of preferred direction (Fig. 4g) and orientation (Fig. 4e) were computed as follows:

$$\Delta Direction = \arccos(\cos(\hat{\mu} - \overline{\mu})),$$

$$\Delta Orientation = \arccos(\cos(2\hat{\mu} - 2\overline{\mu}))\,/2.$$

Spatial tuning

To measure spatial tuning, we presented "on" and "off" (white and black), flashing (300 ms) Gaussian dots. The dots were isotropically shaped, with a s.d. of approximately 8 visual degrees in the centre of the monitor. The position of each dot was randomly sampled from a 17×29 grid tiling the height and width of the monitor. We observed a stronger neuronal

response for “off” compared to “on”, and therefore we used only the “off” Gaussian dots to perform spatial tuning from the in vivo and in silico responses.

To measure spatial tuning, we first computed the STA of the stimulus. Let $x \in \mathbb{R}^2$ denote the spatial location (height and width) in pixels. The value of the STA at location x was computed as follows:

$$\overline{s_x} = \frac{\sum_t |s_{xt} - s_0| r_t}{\sum_t r_t},$$

where r_t is the response of the neuron, s_{xt} is the value of the stimulus at location x and time t, and s_0 is the blank or grey value of the monitor.

To measure the spatial selectivity of a neuron, we computed the covariance matrix or dispersion of the STA. Again using $x \in \mathbb{R}^2$ denote the spatial location (height and width) in pixels:

$$z = \sum_x \overline{s_x},$$

$$\bar{x} = \sum_x \overline{s_x}\, x/z,$$

$$\Sigma_{STA} = \sum_x \overline{s_x}(x - \bar{x})(x - \bar{x})^T / z.$$

The SSI, or strength of spatial tuning, was defined as the negative-log determinant of the covariance matrix:

$$SSI = -\log|\Sigma_{STA}|.$$

To determine the parameters of spatial tuning, we used least squares to fit the STA to the following parametric model:

$$f(x|\boldsymbol{\mu}, \Sigma, \alpha, \gamma) = \alpha \exp\left(-\frac{1}{2}(x - \boldsymbol{\mu})^T \Sigma^{-1}(x - \boldsymbol{\mu})\right) + \gamma,$$

which is a 2D Gaussian component with amplitude α, mean μ, and covariance Σ, plus a baseline offset of γ.

From the in vivo and in silico responses, we estimated two sets of spatial tuning parameters. Let $\hat{\mu}$ and $\overline{\mu}$ be the means (preferred spatial locations) estimated from in vivo

and in silico responses. To measure the difference between the preferred locations (Fig. 4i), we computed the Euclidean distance:

$$\Delta Location = |\hat{\boldsymbol{\mu}} - \boldsymbol{\mu}|.$$

Anatomical predictions from functional weights

To predict brain areas from readout feature weights, we used all functional units in the MICrONS data from 13 scans that had readout feature weights in the model. We trained a classifier to predict brain areas from feature weights using logistic regression with nested cross validation. For each of the 10 folds, 90% of the data was used to train the model with another round of 10 fold cross validation to select the best L2 regularization weight. The best-performing model was used to test on the held-out 10% of data. Finally, all of the predictions were concatenated and used to test the performance of the classifier (balanced accuracy) and generate the confusion matrix. The confusion matrix was normalized such that all rows sum to 1, thus the diagonal values represent the recall of each class.

To predict cell types, the same functional data source was used as in the brain area predictions. Cell types were obtained from CAVEclient initialized with `minnie65_public` and table `aibs_metamodel_mtypes_v661_v2`. To associate a neuron's functional data with its cell type, we merged the cell types to a match table made by combining the manual and fiducial-based automatic coregistration described in MICrONS Consortium et al. cite(MICrONS_Consortium2021-my). Finally, because each neuron could be scanned more than once, and thus could have more than one functional readout weight, we subset the data such that each neuron only had one readout weight according to its highest cc_max. Following this procedure, n=16,561 unique EM neurons remained. Out of the 20 cell classes, all excitatory neuron classes in L2-5 were chosen (except L5NP, which had comparably fewer coregistered cells), leaving 11 classes: L2a, L2b, L2c, L3a, L3b, L4a, L4b, L4c, L5a, L5b, L5ET. To train the classifier using readout weights to predict cell types, logistic regression was used with the same nested cross validation procedure and performance metric as described in the brain area predictions.

For testing whether readout weights contributed to cell-type predictons beyond imaging depth, the 2P depth of each functional unit was obtained from a 2P structural stack (stack session 9, stack idx 19) wherein all imaging planes were registered cite(MICrONS_Consortium2021-my). This provided a common reference frame for all functional units. The two logistic regression models (depth vs depth + readout weights) were trained with all of the data, and the predicted probabilities and coefficients from the

models were used to run the likelihood ratio test, where a P value less than 0.05 was chosen as the threshold for statistical significance.
